# Improved cryopreservation of spermatozoa using vitrification: comparison of cryoprotectants and a novel device for long-term storage

**DOI:** 10.1007/s10815-019-01505-x

**Published:** 2019-07-04

**Authors:** Helen C. O’Neill, Maya Nikoloska, HiuTung Ho, Alpesh Doshi, Walid Maalouf

**Affiliations:** 1 Institute for Women’s Health, UCL, London, UK; 2 Reproductive Medicine Unit, UCLH, London, UK; 3 European Sperm Bank, Copenhagen, Denmark; 4 CRGH, London, UK; 5 University of Nottingham, Nottingham, UK

**Keywords:** Sperm, Vitrification, Slow-freezing, Cryoprotectants

## Abstract

**Study question:**

Does cryoprotection of spermatozoa using a vitrification protocol with improved cryoprotective agents and a novel device for large storage lead to better outcomes than conventional slow freezing?

**Summary answer:**

Vitrification of human sperm using sucrose and dextran-based cryoprotectant (CPA4) with a new vitrification device resulted in significantly better sperm motility and progressive motility and improved DNA integrity with lower DNA fragmentation compared with conventional slow freezing.

**What is known already:**

A major limitation to clinical implementation of vitrification is the right balance between the volume of spermatozoa suspension cryopreserved and a standardised use of CPAs for survival of spermatozoa.

**Study design, size, duration:**

This was a control versus current clinical practice study using 30 fresh human semen samples to carry out the different cryoprotectant analyses followed by a further 23 semen samples to test the novel vitrification protocol.

**Participants/materials, setting, methods:**

All human specimens fulfilled the following criteria: > 5 million spermatozoa/mL, > 20% total motility, ≥ 1.8 mL in volume, with all participants falling within the age range of 25–45 inclusively. The concentration, progressive motility, non-progressive motility, immotility, and various morphokinetic variables including DAP, DCL, DSL, LIN, and STR were then determined using the IVOS II™ Clinical CASA system (Hamilton Thorne, Beverly, MA, USA) on the basis of the 5th Edition of *WHO Laboratory Manual for the Examination and Processing of Human Semen*.

**Main results and the role of chance:**

Among the 6 cryopreservation methods in this study, vitrification with the funnel-shaped device using CPA4 best preserves the 13 sperm parameters evaluated by CASA system. Conventional slow freezing and vitrification with the device using seminal plasma also protects sperm quality, but the overall motilities are statistically lower in comparison with the novel vitrification approach with cryoprotective media using the device. DNA fragmentation significantly increased after cryopreservation through the method of conventional slow freezing (*p* = 0.07). There was no significant difference in DNA fragmentation between fresh control and vitrification (*p* = 1.000).

**Limitations, reasons for caution:**

Extensive training is required to minimise the human error in using the vitrification device to perform cryopreservation. Each operator can only handle one sample at a time with device vitrification, whereas several samples can be processed without the need for special training with conventional slow freezing.

**Wider implications of the findings:**

The presented study shows that a new vitrification method could improve survival sperm rate. Human sperm vitrification using our novel protocol gives higher motility and progression and lower percentage of DNA fragmentation than conventional slow freezing. Our findings indicate that this method could supersede the current clinical practice in particular for patients with oligospermia as it reduces osmotic damage, time, and cost.

## Introduction

Sperm cryopreservation is a core-assisted reproductive technology (ART) procedure for the preservation of male fertility, particularly useful in oncology patients, vasectomy, or other obstructive surgeries, gamete donation programmes, minimisation of sexually transmitted disease transmission, or when male partner is unable to provide an adequate sample on the day of ovum pickup. Since the initial report of human sperm freezing [1], slow freezing, which is a method of stepwise cooling over a period of 2–4 h and involves the use of permeable cryoprotectants, continued to be the most commonly used technique for sperm cryopreservation [2]. However, the long exposure of sperm cells to cryoprotectants can cause physical and chemical damage to the sperm function due to ice crystal formation and osmotic stress [3–5].

Vitrification is now the most popular and more successful method for freezing eggs and embryos in clinical ART laboratories [6]. Vitrification requires higher cooling rates and higher concentrations of cryoprotectants (CPA) in order to bypass the ice formation stage and move into glass solidification instead [7]. However, this method cannot be directly reproduced on spermatozoa [8], due to the high concentrations of permeable CPA which can be damaging to sperm cells. The first reports of successful human sperm vitrification were described by Isachenko and colleagues [9] without cryoprotectants, and again with sucrose [10]. Improvements on the methodology of vitrification included the copper loop approach, where a thin film of sperm suspension was created by dipping the loop into sperm suspension then plunged directly into liquid nitrogen [11]. Other methods such as microdroplets, open-pulled straw (OPS), and open-standard straw systems were also proposed [8]. More recently, a novel sperm vitrification device for small number of spermatozoa was also reported with promising results [12].

The small total volume of spermatozoa suspension cryopreserved by various vitrification systems, such as cryoloops and OPS is another major limitation hindering the routine application of vitrification in clinical settings [13], as the small number of spermatozoa present in such small volume of suspension has seen limited use for IUI, IVF, or other ART procedures apart from ICSI [14, 15]. In addition, limited sperm vitrification systems have been shown to be feasible alternatives [11].

The present study was a twofold investigation, first to compare different compositions of sperm vitrification media and second to compare distinct methods of sperm cryopreservation on post-thaw survival criteria. The initial study compared five vitrification cryoprotectants (CPAs 1-5) using the novel vitrification device and unfrozen samples. Post-thaw sperm parameters were examined comparing the cryoprotective efficacy of different freezing techniques and CPAs using computer-assisted sperm analysis systems (CASA). CPA4 which demonstrated the best survival rate of spermatozoa post-warming was used in the second arm of the study. In the second part of the work, different protocols of sperm cryopreservation were compared, namely conventional slow freezing with cryoprotectant (SFCPA); slow freezing with seminal plasma (SFSEM); direct vitrification in a cryovial with CPA (VITCPA); direct vitrification in a cryovial with seminal plasma (VITSEM); vitrification using a new vitrification device in a cryovial with CPA (VITDCPA); and vitrification using the new vitrification device in a cryovial with seminal plasma (VITDSEM). Conventional slow freezing was carried out as a reference of the performance of current human sperm cryopreservation protocols. Post-thaw survival was assessed using WHO sperm parameters and morphokinetic variables using computer-assisted sperm analysis (CASA) software; DNA damage was measured using a Sperm Chromatin Dispersion (SCD or Halo) test (HALOsperm) and compared between methods to evaluate the most effective way to cryopreserve large volumes of human spermatozoa.

## Materials and methods

### Semen collection

This study was approved by the UCL Research Ethics Committee and the NHS Research Ethics Committee (REC reference, 05/Q0502/143). Written informed consent was obtained from all patients of the Reproductive Medicine Unit in University College London Hospital to participate in this research study. Thirty fresh semen samples were used in the first part of this study to carry out the different cryoprotectant analyses, and this was followed by a further 23 samples to test the novel vitrification protocol. All specimens fulfilled the following criteria: > 5million spermatozoa/mL, > 20% total motility, ≥ 1.8 mL in volume, with all participants falling within the age range of 25–45.

### Obtaining human seminal fluid

A volume of 1 mL of the raw semen sample from the participant’s ejaculate was centrifuged for 10 min at 3500×*g*. The seminal supernatant was aliquoted into a fresh tube without disturbing the pellet to use as cryoprotectants.

### Semen analysis

Sperm parameters were reported after sperm washing (post-prep control), and after thawing/warming. Semen analyses were performed by loading 5 μL of respective samples into the chambers of Leja disposable counting slides (20 μm depth; Leja, Nieuw-Vennep, The Netherlands), where the samples were allowed to fill up the chambers by capillary action. The concentration, progressive motility, non-progressive motility, immotility and various morphokinetic variables including DAP, DCL, DSL, LIN (VSL/VCL), and STR (VSL/VAP) were then determined using the IVOS II™ Clinical CASA system (Hamilton Thorne, Beverly, MA, USA) on the basis of the 5th Edition of *WHO Laboratory Manual for the Examination and Processing of Human Semen*. Post-thaw samples with concentrations lower than 0.20 × 106/mL were however analysed by only assessing 100 sperm. Raw semen samples that were of concentration higher than the operational range of the CASA system (60 × 106/mL) were diluted accordingly with pre-warmed (37 °C) QUINN’s™ Sperm-Washing Medium (gentamicin, 5.0 mg/mL HSA; SAGE; Trumbull, USA).

### Vitrification media

Five vitrification media compositions were supplied by Kitazato BioPharma, Shizuoka, Japan, for evaluation as follows:

- CPA1-0.5 M sucrose with 20% hydroxypropyl cellulose (HPC) (1000 mOsm)
- CPA2-0.5 M sucrose without HPC
- CPA3-0.3 M sucrose with HPC (1000 mOsm)
- CPA4*-0.3 M sucrose with 20% (*v*/*v*) dextran supplement
- CPA5-0.3 M sucrose, 7.5% (*v*/*v*) DMSO and ethylene glycol, and 20% dextran serum supplement.

*From all above-mentioned CPAs, CPA4 gave the best survival rates and was used for reminder of the study.

### Sperm preparation by density gradient centrifugation

Two-layer discontinuous density gradient (45% and 90%) method was employed to isolate spermatozoa and other constituents of semen and to fractionate subpopulations of spermatozoa according to their motility. Forty-five percent and ninety percent of gradient solutions were prepared beforehand by diluting Sperm Preparation Media (MediCult Origio, Berlin, Germany) with sperm-washing medium. A minimum of 1.8 mL of raw semen sample from each participant was prepared and washed with this protocol. First, a sterile Pasteur pipette was used to add 1 mL of the top layer gradient solution (45%) to a centrifuge tube. With a new pipette, 1 mL of the lower layer (90%) gradient solution was then carefully loaded from the bottom of the tube, raising the top layer slowly without disruption to create a sharp interface. A maximum of 1.5 mL of raw semen sample was gently layered on top of the gradient in each tube. Depending on the amount of raw semen sample collected from the patient, multiple gradients were created when necessary. The loaded gradients were later centrifuged at 300*g* for 20 min.

After centrifugation, the supernatant was aspirated in a circular motion from the surface without disrupting the pellet. Three millilitres of pre-warmed (37 °C) QUINN’s™ Sperm-Washing Medium was added to a new sterile tube. Using a Pasteur pipette, the pellet in the centrifuge tube was then aspirated and resuspended in the sperm-washing medium. Next, the mixture was centrifuged for 10 minutes at 300*g*. The supernatant was removed, leaving behind the pellet. Finally, the test wash sample was generated by resuspending the pellet in 1.2 mL of sperm-washing medium and was ready for post-preparation sperm analysis and subsequent cryopreservation procedures.

### Slow freezing

Conventional slow freezing was performed by mixing 200 μL of SpermFreeze™ slow-freezing cryoprotectant (HAS, glycerol, HEPES; FertiPro NV, Beernem, Belgium) drop by drop with the same volume of washed sperm sample in a cryovial. The same procedure was performed for slow freezing in seminal fluid, with participant’s seminal plasma in place of the SFCPA. The mixture was left at in room temperature for 10 min before exposing to liquid nitrogen vapour for 15 min. The sample was then fixed onto a cryocane and plunged directly into liquid nitrogen at − 196 °C.

### Preparation of vitrification solutions

Two hundred microlitres of either the vitrification solution or seminal plasma of the patient was pipetted and mixed drop by drop to 200 μL of post-wash sample, with constant shaking to facilitate thorough mixing of the two. This vitrification cryoprotectant mix was then allowed to sit in room temperature for 2 min, whereas the seminal fluid mix was allowed to sit for 10 min before the subsequent vitrification procedure.

### Vitrification procedure

#### Vitrification using vitrification device

**Fig. 1:**
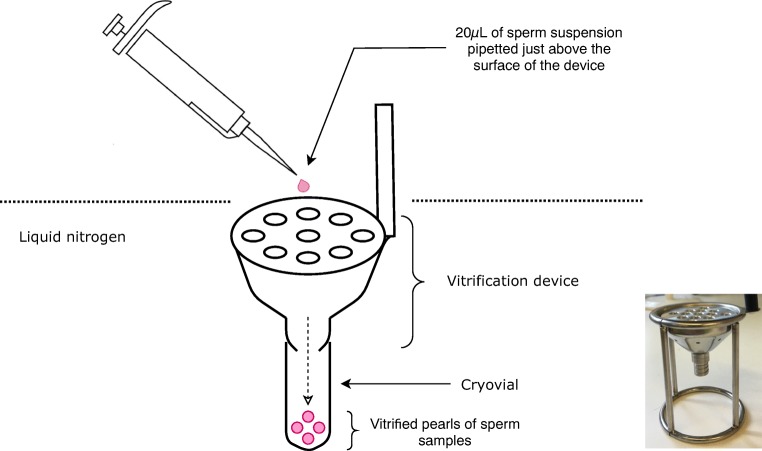
Diagram and photo of the vitrification device and the vitrification process. Twenty microlitres of post-preparation sperm suspension are loaded just above the vitrification device that pre-immersed in liquid nitrogen

A cryovial was screwed to the bottom of the vitrification device provided by Kitazato BioPharma for sample collection during the vitrification process. Under the protection of cryogloves, the vitrification device was grabbed by its plastic handle and slowly immersed into liquid nitrogen held in a foam box, until the metal part was fully submerged and cooled. Twenty microlitres of sperm suspension was rapidly pipetted using a single channel pipette to just above the surface of the metal part of the device without contacting the liquid nitrogen. The pipette was lightly shaken to dispense the droplet of sample formed at the end of the tip. The droplet of sample was vitrified instantly into a pearl at the moment it came into contact with liquid nitrogen that was covering the device. The vitrified pearl was then funnelled through the canals of the device into the cryovial as shown in Fig. 1. After collecting around 10 pearls, the device was removed from the liquid nitrogen, and the cryovial was unscrewed from the device, then capped. The procedure was repeated until all sperm suspension was vitrified, resulting in about 20 pearls in total, stored in 2 separate vials. The vitrification sample was then ready for temporary storage in liquid nitrogen tanks.

#### Vitrification directly into cryovial

After standing in room temperature for the designated time to allow full incorporation of the media or seminal fluid with the sperm samples, the cryovials were plunged directly into liquid nitrogen.

### Thawing and warming

All specimens were thawed after storage for a minimum of 24 h.

#### Slowly frozen samples and those vitrified directly into cryovials

Cryovials of frozen samples were removed from liquid nitrogen and were allowed to thaw at room temperature for 15–20 min until complete transition into liquid state.

#### Samples vitrified with vitrification device

Labelled new round bottom tubes were filled with 2 mL of sperm-washing medium at room temperature prior to warming, each designated for warming vitrified pearls within 1 cryovial. Cryovials containing the vitrified samples were taken out one at a time from liquid nitrogen, and the vitrified pearls were poured one by one into a small sterile tray. The vitrified sperm suspension pearls were quickly picked up by tweezers and dropped into sperm-washing medium one after the other. The tube was shaken lightly to facilitate warming and incorporation of sperm samples into the sperm-washing medium. A new pearl was placed into the sperm-washing medium when the previous one had completely warmed.

### Spermatozoa preparation for analysis after cryopreservation procedures

Each slowly frozen sample and those directly vitrified in a vial were transferred and mixed into 2 mL of sperm-washing medium in fresh tubes after thawing/warming. All frozen/thawed and vitrified/warmed samples were subsequently centrifuged for 10 min at 1200×*g*. The supernatant in each tube was discarded, and the pellet obtained from each cryopreservation approach was later resuspended in 0.5 mL of sperm-washing medium. The warmed and washed sperm samples were then ready for final analysis

### DNA fragmentation analysis

Sperm Chromatin Dispersion (SCD) test (HALOsperm) was used to assess DNA integrity of the samples. The sperm DNA fragmentation assay was performed immediately after obtaining the fresh samples. Fragmentation was then measured after thawing of the two cryopreserved samples. This was carried out according the manufacturer’s instructions (Halotech NDA, Madrid, Spain).

### Statistical analysis

A series of repeated measures ANOVA tests were performed to analyse differences between results obtained from the six different cryopreservation approaches, as well as the difference in initial sperm parameters and post-thaw parameters of each approach. Two-sided Dunnett post hoc tests were subsequently carried out on parameters with statistically significant interactions in ANOVA. All correlation analysis, also the mean and standard deviation (SD) calculations, were performed with SPSS. A *p* value < 0.05 was considered to be statistically significant.

Slow conventional freezing was performed as a reference. The post-thaw sperm parameters of slow freezing were compared against those of vitrification directly in a cryovial, as well as with those resulting from vitrification using a specific vitrification device (Fig. 1) to give an overview of the effectiveness of the proposed novel cryopreservation protocols. All vitrification cycles were performed with our previously evaluated cryoprotective media provided by Kitazato BioPharma (0.3 M sucrose, 20% (*v*/*v*) dextran serum supplement). To evaluate the protective effect of seminal plasma, each of the 3 approaches above were repeated using seminal fluid of the patient as a cryoprotectant instead. Six approaches in total using different cryopreservation methods and cryoprotective media were performed on each consented patient sample. Each raw semen sample was divided into 2 portions, with 1 aliquot ≥ 0.8 mL semen centrifuged to obtain sperm-free seminal plasma, and the rest of the fresh raw semen prepared and washed by density gradient centrifugation. The washed sample was then subdivided into 6 portions, each of 200 μL for examining each cryopreservation approach.

## Results

### The effects of composition of vitrification solutions on sperm survival

Semen samples from 30 different patients were tested (*n* = 30). Each sample was prepared through density gradient, and the semen parameters were assessed for concentration, motility (PM, NP, and IM), and CASA kinetic measurements (DAP, DCL, DSL, LIN, and STR), and the final volume was divided equally and vitrified with one of the five vitrification solutions (CPA1-CPA5). The samples were subsequently warmed, and the semen parameters were reassessed. All CPAs demonstrate similar concentration post-warming (*p* > 0.05) (Table 1). Both motility and progressive motility parameters were significantly higher with CPA4 post-warming compared with all other compositions (Table 1), whilst CPA5 resulted in a significantly lower motility and progressive motility post-warming (Table 1).

**Table 1:** Comparison of spermatozoa survival rates using 5 different cryoprotectants (CPA1-CPA5) to the post-density gradient control. Numbers given are mean ± SD, *p < 0.05, ^+/−^*p* < 0.05 (CPA1 to CPA5 comparison excluding control)

	Control	CPA1	CPA2	CPA3	CPA4	CPA5
Concentration (M/mL)	46.8* (± 61.1)	23 (± 15.9)	15.7 (± 18.0)	17 (± 20.2)	14.2 (± 15.5)	14.4 (± 15.6)
Total motility (PM + NP, %)	82.2* (± 13.8)	17.7 (± 9.4)	18.6 (± 9.2)	21.9 (± 8.9)	31.2^+^ (± 11.4)	2.4^−^ (± 2.1)
Progressive motility (%)	53.2* (± 25.8)	5.6 (± 4.3)	5.2 (± 4.9)	9.8 (± 6.4)	15.5^+^ (± 8.6)	0.8^−^ (± 1.0)
DAP (μm)	15.5* (± 6.7)	11.4 (± 6.0)	10.5 (± 6.2)	14.3* (± 7.9)	14.0* (± 7.7)	10.0 (± 6.4)
DCL (μm)	26.9* (± 12.4)	23.8 (± 11.8)	22.4 (± 12.5)	27.5* (± 13.9)	26.8* (± 13.8)	21.0 (± 13.6)
DSL (μm)	9.7* (± 5.0)	7.9* (± 5.4)	7.1 (± 5.5)	10.7*^+^ (± 7.3)	10.4*^+^ (± 7.1	6.4 (± 4.9)
LIN (VSL/VCL, %)	37.8* (± 16.5)	33.7* (± 16.0)	31.2 (± 16.5)	38.3* (± 16.2)	38.0* (± 16.0)	35.0* (± 19.7)
STR (VSL/VAP, %)	64.9 (± 20.8)	66.4 (± 20.1)	64.6 (± 20.9)	71.8 (± 16.2)	70.3 (± 19.5)	65.1 (± 24.4)

No significance was demonstrated between CPA3, CPA4, and pre-vitrification controls for the majority of CASA kinematic measurements (DAP, DCL, DSL, LIN, and STR, Table 1), which again highlights that CPA4 better post-warming parameters compared with the other vitrification compositions.

### Comparison between seminal fluid and CPA4 vitrification media, with or without the vitrification device, on sperm parameters after vitrification and warming

**Fig. 2:**
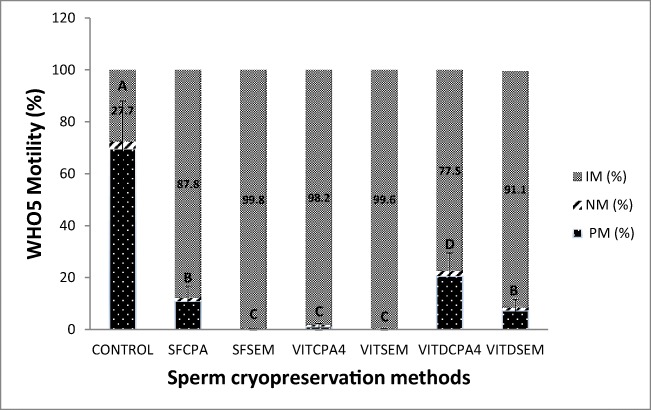
Mean (%) ± SD of PM (progressive motile), NP (non-progressive motile)**,** and IM (**i**mmotile) of post-prep test wash samples prior to freezing and post-thawed samples cryopreserved by the 6 different protocols; different letters between groups indicate statistically significant differences at *p* < 0.05

Sperm parameters of 23 semen samples including concentration and motility (progressive motile (PM), non-progressive motile (NP), and immotile (IM) categories) were assessed, after density gradient centrifugation and prior to freezing or vitrification (post-wash control), and immediately after thawing or warming procedure. VITDCPA4 resulted in a significantly (*p* < 0.05) higher motility parameters post-warming (23%, Fig. 2) than all other post-thaw/warm methods. This was followed by slow freezing (SFCPA, 12%) and VITDSEM (8%) which resulted in significantly higher motility results than VITCPA4 (2%), SFSEM (1%), and VITSEM (1%, *p* < 0.05, Fig. 2).

For SFCPA and VITCPA4 groups, CASA kinematic parameter (DAP, DCL, DSL, LIN, and STR) post-warming were significantly higher than all other treatment groups (data not shown), which again highlights that CPA4 and the novel device results in improved post-warming semen parameters compared with the other vitrification methods.

### Further analysis of slow freezing versus vitrification using the novel vitrification device and CPA4 containing 4. 0.3 M sucrose with 20% (*v*/*v*) dextran serum supplement as the cryoprotectant

A further 23 samples were analysed; concentration, total motility, and progression were found to be significantly lower in the cryopreserved samples when compared with the control (*p* < 0.05, Table 2). Total motility and progressive motility were significantly lower in the slow freezing/thawing group compared with the control and vitrified/warmed samples (Table 1).

**Table 2:** Comparison of semen parameters between control and frozen/thawed or vitrified/warmed samples. Values are expressed as mean ± SD, *^+−^*p* < 0.05

	Sperm concentration	Motility (PM + NP)	Progressive motility (PM)	DNA fragmentation
Control	30.1 ± 28.2*	77.8 ± 18.2*	49.5 ± 23.3*	16.3 ± 12.9
Slow freezing	9.7 ± 11.2	21.2 ± 10.2^−^	7.2 ± 6.2^-^	25.5 ± 17.1*
Vitrification	10.0 ± 13.9	30.1 ± 10.9^+^	16.2 ± 7.4^+^	18.1 ± 12.8

### DNA fragmentation analysis

DNA fragmentation levels were 1.4 times higher in slowly frozen/thawed samples compared with controls and with vitrified warmed samples (*p* < 0.05), whilst there was no significant difference in DNA fragmentation between control and vitrified/warmed samples (Table 2).

## Discussion

The present study was designed to improve on sperm vitrification methods for larger volume samples which remain at a proof of principle stage compared with the conventional slow freezing. We compare five different vitrification solutions that were supplied from a leading manufacturer of vitrification media for gametes and embryos. Vitrification using sucrose and dextran (CPA4) with the new vitrification device resulted in a significantly better sperm motility and progressive motility compared with conventional slow freezing. In the present study, sperm quality that is conventionally estimated by percentage of the total motility was significantly reduced after slow freezing from 77.8 to 21.2% for slow freezing and 30.1% for vitrification (Table 2). The low survival rate is of particular importance in cases where severe oligozoospermia and epididymal or testicular samples are given. The main concern with these patients is the technical difficulties when dealing with such small number of cells, the toxic effects of the CPA’s, and the risk of cross-contamination when an open-freezing system is used [16, 17]. Additionally, vitrification of sperm results in significantly lower DNA damage than with slow freezing as demonstrated by Sperm Chromatin Dispersion analysis. DNA fragmentation significantly increased after cryopreservation through the method of conventional slow freezing (*p* = 0.07). There was no significant difference in DNA fragmentation between fresh control and vitrification (*p* = 1.000).

The average mean DNA fragmentation for slow freezing was found to be 25.5 ± 17.1 (Table 2), which is 1.39 times higher as compared with the vitrification group (18.1 ± 12.8). The lowest DNA fragmentation for slowly frozen samples was 2% and the highest was 62%. However, in the vitrified group, the lowest DNA fragmentation rate was 1% and the highest was 56%.

SPSS assumption of normality gave statistically significant difference (*p* value of 0.41) between DNA fragmentation index of slow freezing and vitrification as shown in Table 2.

These results indicate that vitrification of sperm can be more effective and efficient for sperm cryopreservation in a clinical setting. The cryoprotecting efficacy of sucrose was previously supported by Isachenko et al. reporting a significant increase in progressive motility and viability under supplementation of HSA and sucrose [10]. Sucrose was also reported to also independently successfully preserve motility and viability of sperm among various concentrations of other saccharides in multiple studies. Sucrose along the high molecular weight dextran which has multiple hydroxide groups that may help form a protective layer on the sperm surface by developing hydrogen bonds with the phosphate of the membrane phospholipids.

The purpose of the new funnel vitrification device is to fractionate the sperm suspension into portions of smaller volumes, so as to maximise the contact surface area with liquid nitrogen, thus maximising the temperature drop experienced by each sperm during the vitrification process. Pelleting of sperm has been used successfully in the ram for breeding purposes [18, 19]. Successful vitrification of small volume of semen and the use of vitrification devices were reported previously [10, 20] by aliquoting a suspension of washed semen aliquoted by a single channel pipette into liquid nitrogen and collected by a strainer, also demonstrated superiority of such method over conventional freezing. Rapid cooling rate in liquid nitrogen also greatly shortens the process of change of state of cellular contents [21, 22], effectively avoiding any damage resulting from crystallisation action compared with the 2-stage cooling process in conventional slow freezing [23]. The immersion and complete cooling of the vitrification device in LN2 prior to the start of the procedure minimises the heat insulation effect of film boiling during plunging of the sample into LN2. Film boiling refers to the strong vaporisation process of LN2 upon heat flux during plunging of samples into LN2, resulting in the generation of a layer of vapour covering the LN2 surface adjacent to the heat surface, which reduces thermal conductivity and ultimately the cooling rate.

Despite the superiority of this novel device vitrification approach with vitrification media over conventional slow freezing, the sperm parameters are still greatly diminished in comparison with fresh and washed ejaculate. One limitation of this study would be the warming rate of the pellets which have been demonstrated previously to be a limiting factor in vitrification procedure [13], and increasing the temperature of warming to almost 42 °C resulted in faster thawing past the 0 °C and significantly better survival rates and recovery of motility [24]. It is also worth mentioning that survival of sperm in this work refers solely to motile function so the survival has been potentially under reported as live/dead sperm staining would have highlighted a higher number of sperm survival [10]. Therefore, future studies in the short term should focus on optimising the current vitrification device and media and address the findings in this study with a larger sample size. However, this method could prove superior in the short term for severe male factor infertility where small number of cells are available to freeze.

## Abbreviations

CPA: Cryoprotective agent
DAP: Distance average path
DCL: Distance curved line
DSL: Distance straight line
LIN: Linearity
STR: Straightness
VAP: Average path velocity
VCL: Curvilinear velocity
VSL: Straight-line velocity
